# Wireless Channel Prediction of GRU Based on Experience Replay and Snake Optimizer

**DOI:** 10.3390/s23146270

**Published:** 2023-07-10

**Authors:** Qingli Liu, Peiling Wang, Jiaxu Sun, Rui Li, Yangyang Li

**Affiliations:** 1 Communication and Network Laboratory, Dalian University, Dalian 116622, China; liuqingli@dlu.edu.cn (Q.L.); sunjiaxu@s.dlu.edu.cn (J.S.); lirui@s.dlu.edu.cn (R.L.); liyangyang@s.dlu.edu.cn (Y.L.); 2 School of Information Engineering, Dalian University, Dalian 116622, China

**Keywords:** channel prediction, GRU, snake optimizer, experience replay, online training

## Abstract

Aiming at the problem of poor prediction accuracy of Channel State Information (CSI) caused by fast time-varying channels in wireless communication systems, this paper proposes a gated recurrent network based on experience replay and Snake Optimizer for real-time prediction in real-world non-stationary channels. Firstly, a two-channel prediction model is constructed by gated recurrent unit, which adapts to the real and imaginary parts of CSI. Secondly, we use the Snake Optimizer to find the optimal learning rate and the number of hidden layer elements to build the model. Finally, we utilize the experience pool to store recent historical CSI data for fast learning and complete learning. The simulation results show that, compared with LSTM, BiLSTM, and BiGRU, the gated recurrent network based on experience replay and Snake Optimizer has better performance in the optimization ability and convergence speed. The prediction accuracy of the model is also significantly improved under the dynamic non-stationary environment.

## 1. Introduction

In an epoch of industrial informatization, the 5th Generation Mobile Communication Technology (5G) [1] has become one of the most popular technologies in the world [2]. With the explosive increase in smart terminals, communication systems should meet the needs of more dense, frequent, secure, and efficient user communication. Therefore, 5G uses millimeter wave massive Multiple-Input Multiple-Output (MIMO) to meet user requirements. MIMO will employ antennas in large numbers at the base station and possibly even in user equipment when millimeter wave (mmWave) [3] bands are used [4]. MIMO greatly improves the space utilization and improves the throughput of the communication system. Due to the large-array gain, large-scale MIMO is also considered as a technique to improve the coverage of wireless networks [5]. In MIMO systems, it is usually assumed that channel state information (CSI) is known or estimated, and then used for precoding at the transmitter and detection at the receiver. This is known as the coherent approach. Obtaining CSI is vital to both transmitter and receiver for greater spectral efficiency in the MIMO system. Therefore, the accurate acquisition and prediction of CSI is critical. In addition, some scholars are committed to the study of non-coherent schemes [6,7,8] to reduce the dependence on CSI in communication systems. In this paper, the CSI prediction problem is studied under the coherent scheme.

In large-scale MIMO systems using Time Division Duplex (TDD) mode [9], the transmitter needs to obtain accurate CSI for the pre-coding operation to ensure the signal quality received by the receiver [10]. At present, the wireless communication systems required rely on channel estimation methods to estimate CSI. Channel estimation is the process of estimating the characteristics of the channel by using the various states of the received signal, such as least squares channel estimation, minimum mean square error channel estimation, etc. Then, the adaptive technology automatically adjusts the coding modulation scheme according to CSI to improve the performance and quality of the communication system [11]. Due to the processing delay of the receiver and the transmission delay of the channel, the CSI received by the sender is inconsistent with the current real channel. As a result, large-scale MIMO [12] cannot maximize the throughput of the communication system. Channel prediction technology can predict the CSI of future time in advance, so it can solve the problem of low throughput of communication systems caused by outdated CSI. Channel prediction is very useful in this case. Thus, channel prediction technology is considered one of the effective methods to efficiently acquire CSI in the scene of the fast time-varying channel [13].

In recent years, machine learning has developed rapidly, especially deep-learning algorithms. Among them, time series analysis has become a popular research area [14], and it is also widely used to forecast future outcomes based on recorded historical data. This has generated a lot of interest in the communications field to adopt deep-learning techniques to address communication challenges. In treating highly nonlinear features, deep-learning approaches automatically process via a cascade of multiple layers, in which the Back-Propagation Neural Network (BPNN) and the Recurrent Neural Network (RNN) are two popular algorithms used for predicting time series [15].

In the field of channel prediction, scholars have used the BPNN and the RNN to propose many algorithms. Neural networks utilize a large known data set to find the internal relation, instead of deriving equations based on assumptions and propagation models. Large predictive models experience significant performance degradation when encountering propagation channels that differ from the training set. The complexity of large network models hinders the real-time response required for wireless communication [16].

In this paper, we propose a gated recurrent unit (GRU) based on experience replay [17] and snake optimizer [18] (SO) (ERSO-GRU). The ERSO-GRU model uses the SO algorithm to find the optimal parameters and uses the ER mechanism to regularly train the two-channel GRU network to adapt to the constantly changing non-stationary channels, so that faster and more accurate prediction values can be obtained in the real environment. Firstly, a two-channel prediction structure is designed using the GRU network to predict the real and imaginary parts of CSI. Secondly, the previous experience is used to generate a set of candidate solutions within a certain range, each corresponding to the ERSO-GRU learning rate and the number of hidden layer units. Then, the snake optimization algorithm is used to solve the optimal solution set, and the optimal parameters are used to construct the prediction model. The data pool mechanism is introduced in the online prediction stage. By updating the data in the data pool and using the data in the data pool for online training which can adapt to the continuously changing non-stationary channel, error accumulation is avoided, and the high prediction accuracy of the model is maintained, because the model does not need to realize prediction channel parameters and the introduction of the experience replay can greatly improve the generalization ability and prediction accuracy of the model.

## 2. Related Work

In MIMO systems, there are coherent methods and non-coherent methods. Since the estimation and prediction of a large number of CSIs is difficult, the researchers propose an alternative solution: non-coherent detection. In the field of coherent method channel prediction, researchers have been working to improve the accuracy of prediction. The rapid development of time-series analysis in the field of deep learning has greatly improved prediction technology. Researchers can use neural networks to capture nonlinear relationships in the training data set to obtain more accurate predictions based on the inputs.

### 2.1. Non-Coherent Approach

In the field of incoherent methods, most research has been focused on the constellation design to multiplex and non-coherently detect several users [7]. In [6], a new incoherent multi-access channel joint constellation design scheme is proposed, which reduces the joint symbol error rate at the same transmission rate and power, but this design is only applicable to the symmetric power of two users. In [7], it is proposed to group users who experience Rayleigh fading and users with Rayleigh fading, which reduces the complexity of the receiver but only considers the uplink in the communication system. In [8], two constellation design schemes are designed by using Gaussian approximation optimization and Monte Carlo optimization. Although the bit error rate is reduced, the generalization ability needs to be improved. These incoherent methods can be used to detect special problems and improve the quality of the communication system. However, the generalization ability of these schemes is weak, and the operands at the receiving end are very complex. Therefore, the problem of channel prediction in a coherent scheme is studied in this paper, and an ERSO-GRU model is proposed. The designed structure is experimented and validated on publicly reported real channel measurement data.

### 2.2. Channel Prediction in Coherent Approach

A neural network is a system that processes data in a way that mimics the way biological neurons process information. The system is trained to modify the weights between individual neurons to describe the relationship between the data. In the field of channel prediction, researchers have proposed many algorithms combined with neural networks. The literature [19] presents a multi-time channel prediction system based on BPNN with multi-hidden layers. Meanwhile, an early stopping strategy to avoid the overfitting of BPNN is introduced. BPNN has some shortcomings, such as the final training result, which can easily fall into a local extremum, and cannot remember historical information. RNN can remember historical information, so researchers began using RNN for channel prediction. Literature [20] proposes a channel prediction method based on RNN, which inputs the entire vectorization of the channel matrix into the prediction model for channel prediction. RNN fails to maintain the long temporal dependence because of the serious vanishing/exploding gradient problem [21].

To alleviate these problems, a named long-term memory network (LSTM) is proposed. Therefore, many researchers use LSTM neural networks for channel prediction at present. In [16], based on LSTM neural network, an adaptive parameter less recursive neural structure is proposed for wireless channel prediction, and the method realizes CSI prediction for future moments by conducting offline training and online prediction for CSI of historical moments. In [22], a channel prediction model based on a single-layer LSTM neural network is proposed, and the method is to forecast time-varying channels by sending the channel state information obtained from continuous sampling into the model as input. In [23], based on [22], a channel prediction model based on a double-layer LSTM neural network is proposed. In [24], a new prediction method using the LSTM neural network is proposed, and the method uses the pilot signal received by the predictor to learn the channel change and predict the future channel state. Based on LSTM, scholars put forward GRU, which has fewer parameters and less computation and has the same excellent time series prediction ability. Therefore, this paper proposes the construction of a prediction model using GRU.

## 3. System Model

### Communication Model

**Figure 1:**
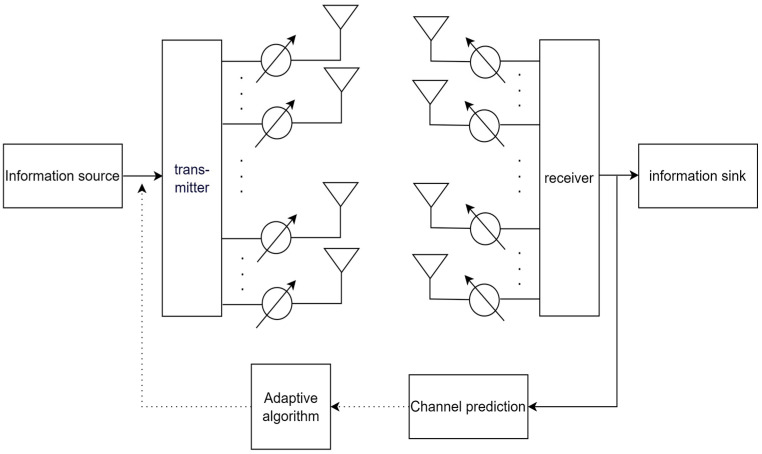
Communication system model diagram.

Figure 1 shows the model diagram of the general point-to-point MIMO system consisting of a single transmitter and a single receiver. This paper mainly improves the channel prediction module in the system and puts forward new solutions.

When the number of transmitting antennas $N_{t}$ and receiving antennas $N_{r}$ is one, the MIMO system becomes a single input single output (SISO) system [25]. In the SISO system, the relationship between the source symbols and the corresponding received signals is shown in Equation (1):

(1) y(t)=h(t)x(t)+z(t)

where x(t) represents the symbols transmitted at the transmitter side, y(t) represents the signals received by the receiver side, h(t) represents the complex CSI, and z(t) is the additive white Gaussian noise at time t.

MIMO systems are similar to SISO systems. As shown in Figure 1, this system is equipped with $N_{t}$ transmitting antennas at the transmitting end and $N_{r}$ receiving antennas at the receiving end. The relationship between the symbols transmitted at the transmitter side and the signals of the corresponding receiving end is shown below in Equation (2),

(2) $${\left(\begin{array}{l} y_{1}(t)\\ y_{2}(t)\\ \vdots \\ y_{N_{r}}(t) \end{array}\right)}_{N_{r}\times 1}={\left[\begin{array}{cccc} h_{11} & h_{12} & \cdots & h_{1N_{t}}\\ h_{21} & h_{22} & \cdots & h_{2N_{t}}\\ \vdots & \vdots & \ddots & \vdots \\ h_{N_{r}1} & h_{N_{r}2} & \cdots & h_{N_{r}N_{t}} \end{array}\right]}_{N_{r}\times N_{t}}{\left(\begin{array}{l} x_{1}(t)\\ x_{2}(t)\\ \vdots \\ x_{N_{t}}(t) \end{array}\right)}_{N_{t}\times 1}+{\left(\begin{array}{l} z_{1}(t)\\ z_{2}(t)\\ \vdots \\ z_{N_{r}}(t) \end{array}\right)}_{N_{r}\times 1}$$

where ${\left[x_{1}(t),x_{2}(t),\cdots ,x_{N_{t}}(t)\right]}^{T}$ represents the transmitting signal, and ${\left[y_{1}(t),y_{2}(t),\cdots ,y_{N_{r}}(t)\right]}^{T}$ represents the signal of the receiving side. The channel state matrix h(t) is shown as follows:

(3) $$h(t)={\left[\begin{array}{cccc} h_{11} & h_{12} & \cdots & h_{1N_{t}}\\ h_{21} & h_{22} & \cdots & h_{2N_{t}}\\ \vdots & \vdots & \ddots & \vdots \\ h_{N_{r}1} & h_{N_{r}2} & \cdots & h_{N_{r}N_{t}} \end{array}\right]}_{N_{r}\times N_{t}}$$

Equation (3), $h_{ij}(i=1,2,\ldots ,N_{t};j=1,2,\ldots ,N_{r})$ represents the channel state information between the *i*th transmitting antenna and the *j*th receiving antenna. To adapts to input of neural network, the channel matrix h(t) vector needs to be transformed into the vector of $1\times N_{r}N_{t}$.

To obtain channel state information, the known pilot symbol p(t) can be sent. The measured value of CSI can be derived from the received signal $\hat{h}(t)$, as shown in Equation (4) [16].

(4) $$\hat{h}(t)=\frac{y(t)}{p(t)}=h(t)+\frac{z(t)}{p(t)}=h(t)+n(t)$$

In this paper, channel prediction based on the pilot signal is considered. In the time-division duplex system, the receiver can use the reverse link signal for CSI estimation and predict the state information of the channel for transmission at the next moment. Assuming that the measured CSI is known at the first P time steps, the system predicts the CSI at the next R time steps. In this work, we find that instead of predicting the CSI directly, predicting the Real and Imaginary parts of CSI leads to better performance separately.

CSI prediction is a time series prediction problem. Thus, this paper proposes to use convolutional neural networks to solve the problem, and a snake optimization algorithm is used to obtain the optimal number of hidden layer units and learning rate of GRU, then use the optimal parameters to construct a ERSO-GRU network.

## 4. SO Improved GRU Model

### 4.1. Gated Recurrent Neural Network

**Figure 2:**
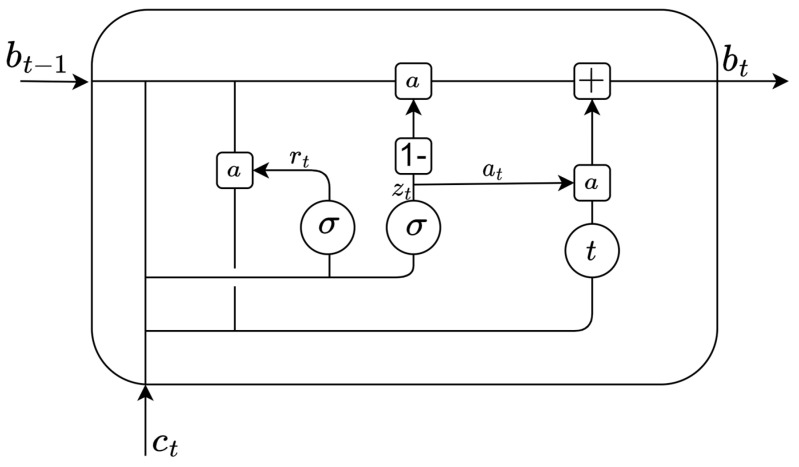
Structure of a gated recurrent unit.

A Gated Recurrent Neural Network (GRNN) is a kind of recurrent convolutional neural network. In GRNN, the GRU [26] is the most common. The internal structure of a Gated Recurrent Unit (GRU) [27] is shown in Figure 2.

According to Figure 2, the equations [21] of GRU can be given as:

(5) $$z_{t}=\sigma (w_{z}[b_{t-1},c_{t}]+b_{z})$$

(6) $$r_{t}=\sigma (w_{r}[b_{t-1},c_{t}]+b_{r})$$

(7) $$a_{t}=t(r_{t}*w_{a}[b_{t-1},c_{t}]+b_{a})$$

(8) $$b_{t}=(1-z_{t})*a_{t}+z_{t}*b_{t-1}$$

where σ is the sigmoid activation function, t is the tanh activation function, $c_{t}$ is the current CSI input, $b_{t-1}$ is the previous CSI input, and $b_{t}$ is the current output. $z_{t}$ and $r_{t}$ represent the update and the reset gates, respectively. $a_{t}$ is the candidate activation.

**Figure 3:**
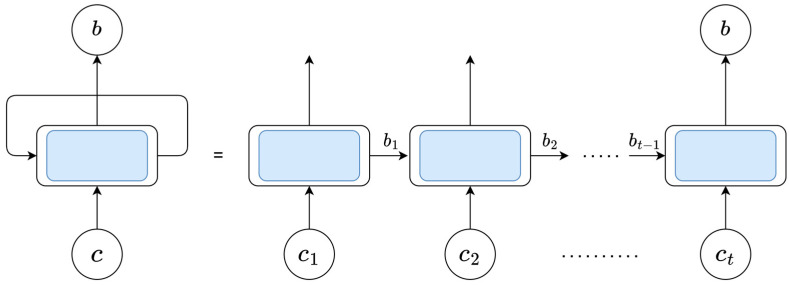
GRU structure diagram.

First, compared with LSTM, GRU only contains an update gate and reset gate, so the corresponding parameters are less than LSTM, the calculation speed is faster, and the efficiency is higher. Secondly, the existence of a feedback loop in the GRU unit enables the neurons in the hidden layer to be connected circularly, which can well capture the dependency relationships with large intervals in the time series data. Therefore, GRU is particularly good at learning the correlation in time series, and there is a close time correlation between each channel state information sequence, so GRU can be used to solve the channel prediction problem. To sum up, GRU is selected in this paper to predict CSI. The complete GRU structure is shown in Figure 3.

Where $c=\{c_{1},c_{2},\ldots ,c_{t}\}$ is the current CSI input. After processing by a GRU unit, the hidden state (which contains the relevant information of the previous node) is passed to the next GRU unit. After receiving the current input and the hidden state information of the previous node, the information is passed to the next node through GRU calculation. To determine the optimal number of GRU nodes, the SO algorithm is adopted in this paper.

### 4.2. SO Improved GRU Prediction Model

**Figure 4:**
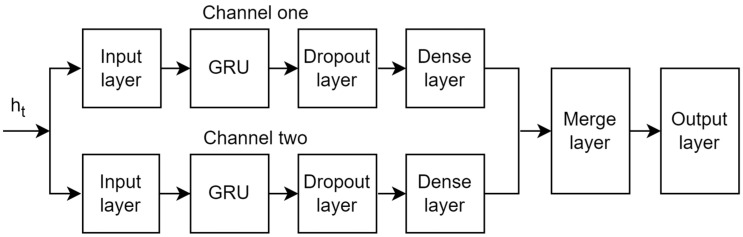
Network model of the prediction model.

Channel prediction is to predict the channel state information at the next time steps according to the recent historical channel state data, and the channel state information is a matrix composed of complex numbers. To improve the accuracy of prediction, this paper uses GRU to construct a channel prediction model with double input and a single output. Its network structure is shown in Figure 4. Channel one and channel two are composed of the input layer, GRU, dropout layer, and dense layer [21], respectively. Then, data from channel one and channel two are fused and finally the predicted CSI is output through the output layer.

Since the CSI matrix is complex, to fully learn the correlation between data, a two-channel prediction model is designed in this paper. The main prediction idea of this model is as follows: in channel one, the input sequence of the CSI real part matrix is fully extracted with bidirectional time dependence using the GRU network, and then the data are obtained through the dropout layer and dense layer. At the same time, the correlation of the CSI imaginary part sequence is extracted in channel two, and then the data are obtained through the dropout layer and dense layer. The purpose of introducing dropout layers in the two channels is to temporarily remove the neural network training unit from the network according to a certain probability to prevent overfitting of the model. The predicted data are obtained in the dense layer of each channel, and the data are integrated through the fusion layer. Finally, the complete CSI prediction data are output through the output layer.

When determining the number of GRU hidden layer units and learning rate, this paper uses a snake optimization algorithm to find the best-hidden layer units and learning rate, to build the optimal network model structure and improve the convergence rate of the model.

### 4.3. GRU Algorithm Improved by SO

When constructing the network model, first determine the boundary range of the number of hidden layer units and the learning rate of the model, and then use Equation (9) [18] to initialize the population.

(9) $$M_{i}=M_{\min}+r\times (M_{\max}-M_{\min})$$

where $M_{i}$ is the position of the i individual, $M_{\min}$ and $M_{\max}$ is the minimum boundary value and maximum boundary value of the number of hidden layer units and learning rate, respectively, and *r* is the random number between [0, 1]. Each individual is composed of the number of hidden layer units one, the number of hidden layer unit two, and the learning rate.

Then, Equations (10) [18] and (11) [18] were used to divide the population into the female population and the male population, and each individual was brought into the network model to calculate the loss value as the fitness of each individual. The best fitness individual for females is $f_{best,f}$, the best fitness position for males is $f_{best,m}$, and the best fitness position globally $f_{food}$.

(10) $$N_{m}=N/2$$

(11) $$N_{f}=N-N_{m}$$

where *N* is the population number, $N_{m}$ refers to the number of the male population, and $N_{f}$ refers to the number of the female population.

**Figure 5:**
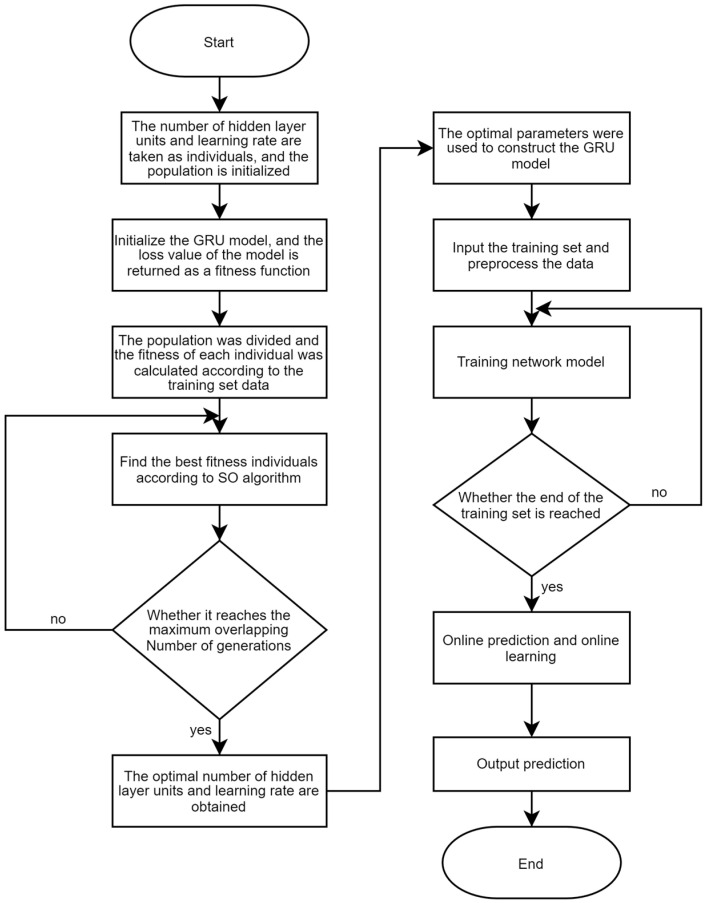
Flow chart of SO-improved GRU algorithm.

Then, this paper uses the SO [18] algorithm to calculate the best parameters of GRU in channel one and channel two. The range of parameter variation was determined according to historical CSI data and inherent requirements of the model, and the SO algorithm was used to find the optimal number of hidden layer units’ neurons_1 and neurons_2 in the given range, as well as the learning rate lr. The flow chart of the GRU algorithm improved by SO is shown in Figure 5:

The detailed algorithm steps are as follows:

Step 1: Build a GRU network model and determine the boundary values of the number of hidden layer units and learning rate according to historical data and inherent model requirements.
Step 2: Import the training set and preprocess the data.
Step 3: Determine the number of iterations according to historical experience, initialize the population with boundary values, and divide the female and male populations.
Step 4: The fitness of everyone is obtained according to the model and training set. The loss value of the network model is used as the fitness function value.
Step 5: Use the SO algorithm to find the best individual iteration.
Step 6: Judge whether the SO algorithm reaches the upper limit of iteration times. If yes, retain the final optimal fitness individual and return the optimal number of hidden layer units and learning rate; otherwise, the operation of Step 5 is repeated.
Step 7: Establish the GRU model with the best parameters, input the pre-processed training set data, and train the network model.
Step 8: Judge whether it reaches the end of the training set data. If yes, proceed to Step 9; otherwise, continue the training.
Step 9: Use the trained network model to conduct online prediction and online training.
Step 10: Output the predicted channel status information.

### 4.4. Model Training and Prediction

**Figure 6:**
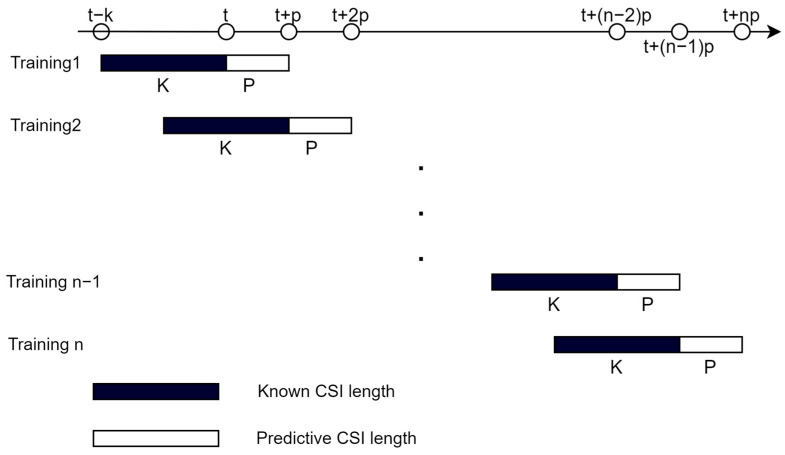
Sequence arrangement table of data block training.

In the offline training stage, the same size of the data block as the online prediction is adopted to train the model. Each data block is composed of several training sequences, and the training set is composed of several data blocks. When training the model, the length of each training sequence is D, where the length of known CSI data is K and the length of prediction is P; that is, D = K + P. Assuming a total of n sequences of data blocks, the training arrangement of a data block is shown in Figure 6. The data block of each training set will be input into the model for learning, to improve the prediction accuracy of the model.

In this paper, the model trains the network by minimizing the difference between the output of the network model and the actual value. In this paper, Mean Square Error (MSE) [20] given by the following equation is selected to continuously optimize the model:

(12) $$MSE=\frac{1}{P}\sum_{j=1}^{P}||\hat{h}[t+j]-h[t+j]|{|}^{2}$$

where h[t+j] is CSI at the t+j time steps in the data set, $\hat{h}[t+j]$ is CSI predicted at the t+j time steps, and *P* is the prediction length.

**Figure 7:**
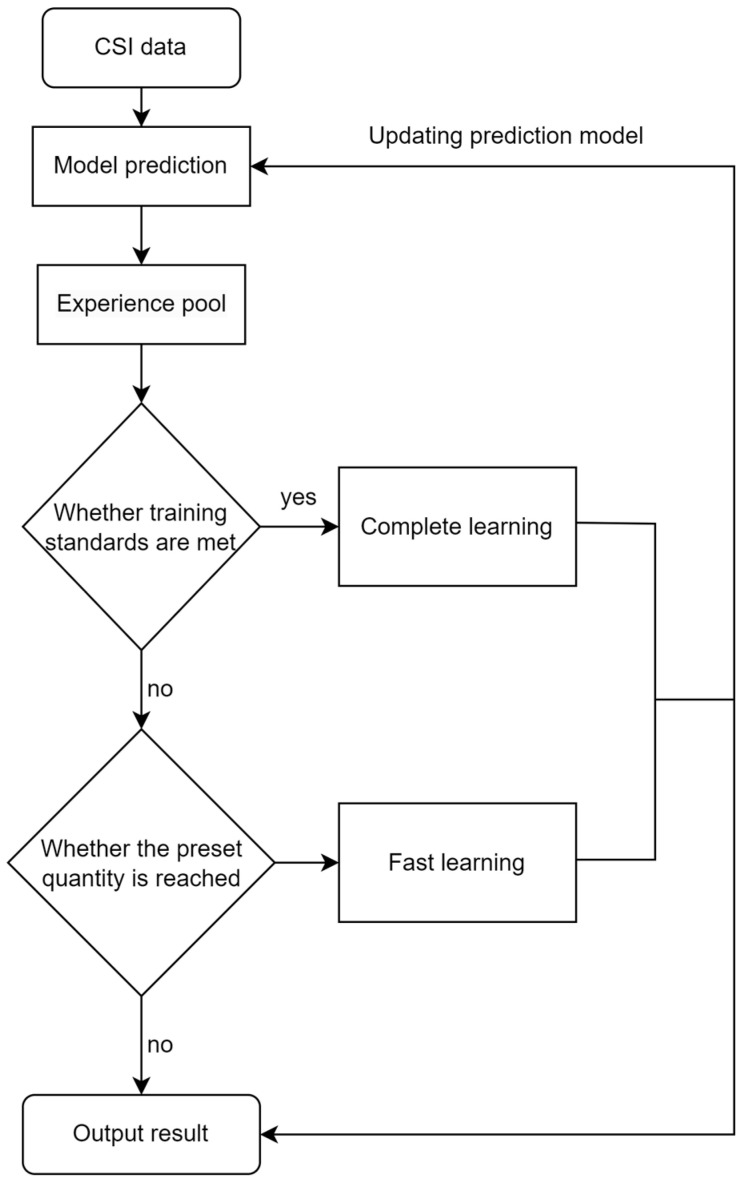
Flow chart of online CSI prediction algorithm based on experience replay.

In this paper, the prediction phase adopts the same sequence arrangement as the training phase, using K known CSIs obtained from the actual communication system to predict P unknown CSIs in the future. In this paper, the experience pool mechanism is introduced in the prediction stage, in order to reduce the accumulation of prediction errors in the neural network model and improve the prediction accuracy. The size of the experience pool is kept consistent with the size of the data block, and its real CSI is stored in the data pool after each sequence of prediction. When the number of predictions is equal to the set parameter h, the system fetches the experience pool data for fast learning, which has adjusted the prediction model; when the experience pool data are all updated, the algorithm will fetch the data for full learning to optimize the prediction network model. Fast learning is performed when the data in the data pool is updated to a certain extent, and full learning is performed when the data pool is completely updated. Both use the same length of data, but the number of times the model is trained is different. The flow of the online prediction algorithm is shown in Figure 7:

Using snake optimizer and empirical playback strategy, the proposed prediction model is summarized in Algorithm 1.
**Algorithm 1:** Proposed channel predictor1. Initialize parameters in the SO, such as population M, iteration times T, etc.2. Use SO to find the optimal parameters.3. The two-channel GRU model was constructed using the optimal parameters.4. Start offline Training.5. **While** True **do**6.  Data preprocessing7.  **if** Total forecast data length > Total data length **then**8.   **end while**9.  **else**10.    i ← 111.    **while** True **do**12.   **if** Forecast data length > D **then**13.      **end while**14.   **end if**15.   Single prediction.16.   Store experience in the experience pool.17.   **if** i % 20 = 0 **then**18.    Complete learning.19.   **else**20.    **if** i % 20 = 0 **then**21.     Fast learning22.   **end if**23.    i ← i + 124.  **end if**

## 5. Analysis of Simulation Results

### 5.1. Data Analysis

The simulation verification of the method proposed in this paper adopts the measured channel values in two wireless environments. The first is the measured data of wireless systems in an industrial environment in the National Institute of Standards and T (NIST) [28]. The NIST data length is 40,500 timestamps. The second data set adopts the measurement values of indoor wireless channels with a frequency of 2.4 GHz provided by Mohamed AlHajri and Nazar Ali in the machine learning library of the University of California, Irvine (UCI) [29]. The UCI measurement divides the measurement area into 196 points with 10 measurements at each point, each of which has a length of 600 timestamps.

### 5.2. Model Parameter Setting and Evaluation Index

All the experiments are performed on AMD Ryzen 7 5800 H with Radeon Graphics 3.20 GHz ThinkBook 14p Gen 2 with 16G RAM running Windows 10 by using Python 3.9.5, Keras 2.9.0 and TensorFlow 2.9.1. To ensure that the SO algorithm can achieve the fastest convergence speed and guarantee the prediction accuracy of the GRU model, the following parameter settings are selected in this paper.

The parameter Settings of the GRU network model improved by ERSO are shown in Table 1:

To evaluate the advantages and disadvantages of the proposed prediction model and other reference methods, we used MAE, MAPE, MSE, and RMSE [27] to evaluate the prediction accuracy of CSI parameters. The formula of each evaluation index is shown in Table 2.

### 5.3. ERSO Improved GRU Algorithm Performance Analysis

To verify the performance of predicting CSI in this study of experience replay and the SO-improved GRU algorithm (ERSO-GRU), the evaluation criteria are used to compare the ERSO-GRU, LSTM, BiGRU, and BiLSTM. We demonstrate the ability of the proposed method by testing it with the measurement data found on site [29]. This paper selects four scenarios in the data set. Lab139 (highly cluttered) and Corridor_rm155 (medium cluttered) [with wall from one side and windows from the other side], Mai-n_Lobby (low cluttered), and Sports_Hall (open space), and then one measurement datum is randomly selected from 10 measurements of 196 measurement points for the experiment. During the experiment, the data were tested 100 times to avoid the existence of special conditions. The results indicate that ERSO-GRU not only works well with the simulation data but also improves the evaluation criteria. The average value of the evaluation criteria of its test results is shown in Table 3:

Table 3 shows the prediction result of the proposed ERSO-GRU and the other methods, using Lab139, Corridor_rm155, Main_Lobby and Sports_Hall indoor channel measurement provided in UCI Machine Learning Repository, of which the length is 600 timestamps. The parameters of ERSO-GRU are the same as those of other methods; only the number of hidden layer units and learning rate of the model are different, and LSTM, BiLSTM, and BiGRU do not adopt the online experience pool mechanism.

It can be seen from the simulation results that the ERSO-GRU algorithm proposed in this paper has a more accurate predictive value, which is improved to a certain extent compared with the other three methods. This is because this paper uses the experience pool mechanism, which can reduce the accumulation of prediction errors in the process of prediction and improve prediction performance. More specifically, in the prediction process, when the number of prediction sequences reaches a certain level, the model will call the data pool data for quick learning. When the data storage of the data pool reaches the upper limit, the prediction model will carry out comprehensive learning to optimize the prediction model and improve the prediction accuracy.

**Figure 8:**
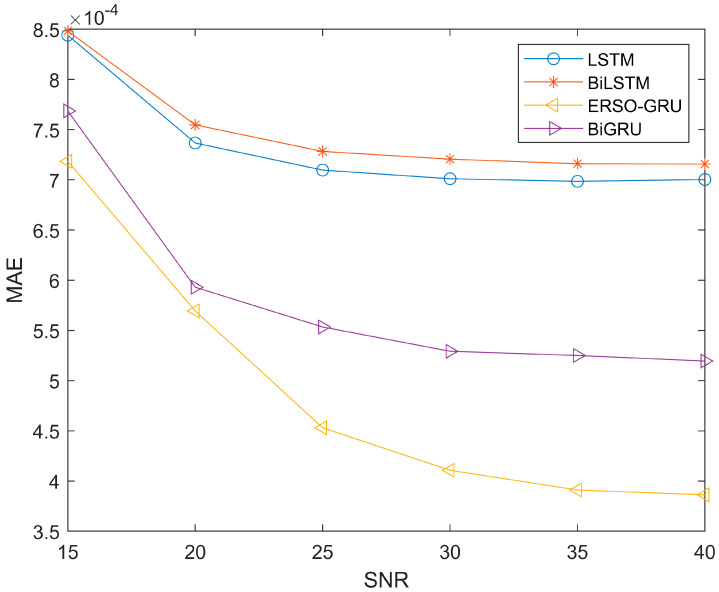
Performance comparison—MAE, with SNR changing from 15 dB to 40 dB.

**Figure 9:**
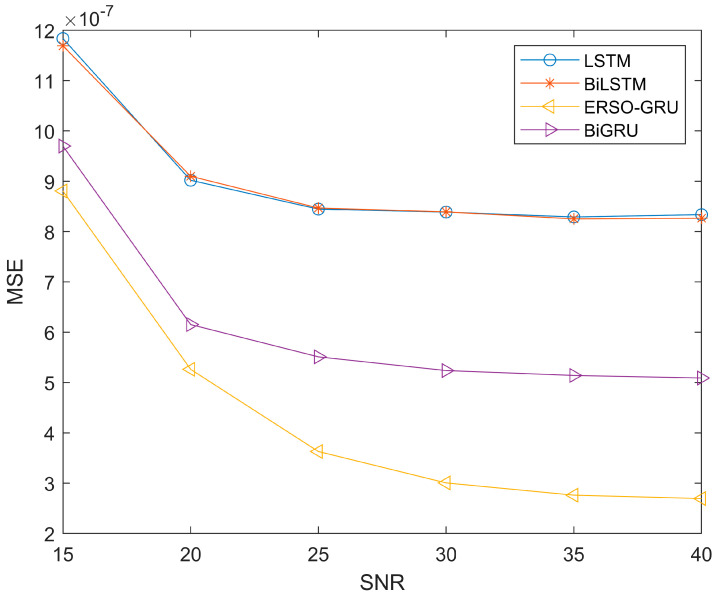
Performance comparison—MSE, with SNR changing from 15 dB to 40 dB.

**Figure 10:**
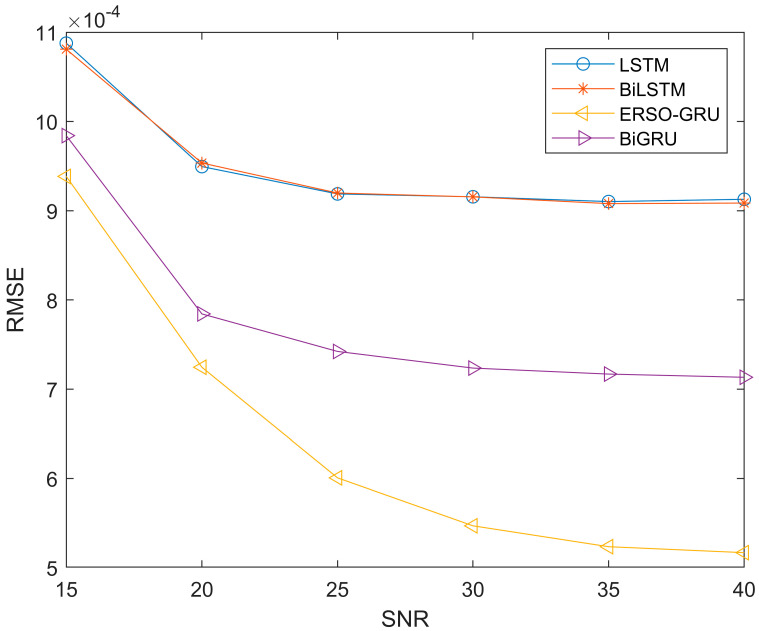
Performance comparison—RMSE, with SNR changing from 15 dB to 40 dB.

Due to the time-varying and multipath nature of the environment, the signal noise ratio will also change during signal transmission. So, to further compare with other methods, more measured channel data are used to verify the performance of the four methods. We use data from the UCI and add Gaussian white noise to measure the impact of signal-to-noise ratio on ERSO-GRU. The signal-to-noise ratio ranges from 15dB-40dB. The parameters of ERSO-GRU and LSTM, BiLSTM, and BiGRU models are the same except for the number of hidden layer units and the learning rate. However, the online experience pool mechanism proposed in this paper is unique to ERSO-GRU. To avoid the particularity of a certain experimental result, this paper repeated this group of experiments 100 times and took the average value of its evaluation criteria. Figure 8, Figure 9 and Figure 10 show the experimental results.

Since the experiment was repeated 100 times and the average values of MAE, MSE and RMSE were obtained, the same type of results was presented. From Figure 8, Figure 9 and Figure 10, throughout the process of increasing the SNR, the ERSO-GRU algorithm outperforms LSTM, BiLSTM, and BiGRU on MAE, MSE, and RMSE. From Figure 8, Figure 9 and Figure 10, we can see that the MAE, MSE, and RMSE drop dramatically when the SNR increases from 15–30 dB, and then keeps decreasing; however, the improvement is becoming slow. As the SNR becomes sufficiently large, there is a diminishing return in terms of MAE, MSE, and RMSE, reaching an MAE, MSE, and RMSE floor. This is expected, as there is always prediction error even when the history CSIs are known perfectly.

To verify the convergence speed and optimization speed of ERSO-GRU, data in NIST was used for experimental comparison of convergence curves with LSTM, BiLSTM, and BiGRU. The convergence curve was obtained from the offline training stage in the training model process. Among them, ERSO-GRU uses Snake Optimizer to find the optimal number of hidden layer units and learning rate.

**Figure 11:**
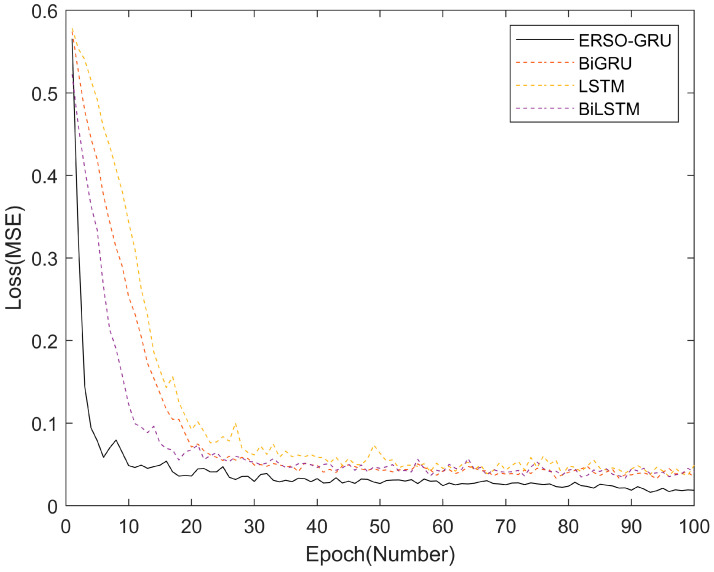
Comparison of convergence curves of the four algorithms.

It can be seen from Figure 11 that the ERSO-GRU algorithm is superior to the other three algorithms, because ERSO-GRU finds the optimal number within the specified range according to the SO algorithm and SCI data characteristics. Therefore, RESO-GRU has a faster convergence rate and a lower loss value than BiLSTM, LSTM, and BiGRU.

## 6. Conclusions

In this paper, we propose a wireless channel prediction model based on experience replay and snake optimizer, which is used to solve the problem of poor prediction accuracy of channel state information in wireless communication systems. The simulation results show that compared with the prediction models based on LSTM, BiLSTM, and BiGRU, the proposed method has a certain degree of improvement on MAE, MSE, and RMSE under different signal-to-noise ratios and has a higher CSI prediction accuracy. Through testing on different real channel data sets, by testing different real channel data sets, the algorithm can maintain high prediction accuracy while having high convergence speed. The future direction of the research is to develop models with an even higher accuracy and even higher speeds to improve the prediction accuracy of the model at low SNR. We can further improve these results by taking into account other external factors such as standardized Doppler shift and base station angle parameters.

## Figures and Tables

**Table 1:** Parameter Settings of GRU network model improved by ERSO.

ERSO-GRU	Value Range
Snake populations M	10
Maximum iterations T	100
Hidden layer units one	[1, 50]
Hidden layer units two	[1, 50]
Learning rate	[0.00001, 0.5]
Known CSI length K	30
Predicted CSI length P	10
Data block size	200
Experience pool size	200

**Table 2:** Model evaluation criteria.

Evaluation Criteria	Definition	Formula
MAE	Mean absolute error	$MAE=\frac{1}{P}\sum_{j=1}^{P}\left||\hat{h}[t+j]-h[t+j]|\right|$
MAPE	Mean absolute percentage error	$MAPE=\frac{1}{P}\sum_{j=1}^{P}\frac{\left||\hat{h}[t+j]-h[t+j]|\right|}{h[t+j]}$
MSE	Mean square error	$MSE=\frac{1}{P}\sum_{j=1}^{P}||\hat{h}[t+j]-h[t+j]|{|}^{2}$
RMSE	Root mean square error	$RMSE=\sqrt{\frac{1}{P}\sum_{j=1}^{P}||\hat{h}[t+j]-h[t+j]|{|}^{2}}$

where P is the size of the prediction length of CSI data, h[t+j] is the observed value (real value) of CSI in the data set on t+j time steps, and $\hat{h}[t+j]$ is the predicted value of CSI data. If the results of MAE, MAPE, MSE, and RMSE are closer to 0, the prediction accuracy of the model is higher.

**Table 3:** Predictive performance of different models.

Locations	Model	MAE	MAPE	MSE	RMSE
Lab139	LSTM	5.9804 × 10^−4^	1.3983	6.6456 × 10^−7^	8.1437 × 10^−4^
	BiLSTM	6.8622 × 10^−4^	1.6185	8.1333 × 10^−7^	8.9885 × 10^−4^
	BiGRU	4.7453 × 10^−4^	1.1571	4.1525 × 10^−7^	6.4409 × 10^−4^
	ERSO-GRU	3.4329 × 10^−4^	0.7859	2.5012 × 10^−7^	4.9732 × 10^−4^
Corridor_rm155	LSTM	6.8724 × 10^−4^	1.4415	7.1536 × 10^−7^	8.4559 × 10^−4^
	BiLSTM	7.0936 × 10^−4^	1.5649	7.5727 × 10^−7^	8.6966 × 10^−4^
	BiGRU	4.7949 × 10^−4^	0.7473	3.8292 × 10^−7^	6.1840 × 10^−4^
	ERSO-GRU	4.0665 × 10^−4^	0.6846	3.1759 × 10^−7^	5.6068 × 10^−4^
Main_Lobby	LSTM	6.1129 × 10^−4^	4.0924	6.4957 × 10^−7^	8.0544 × 10^−4^
	BiLSTM	6.8232 × 10^−4^	5.7196	8.1423 × 10^−7^	9.0118 × 10^−4^
	BiGRU	4.3570 × 10^−4^	2.8235	3.5520 × 10^−7^	5.9579 × 10^−4^
	ERSO-GRU	3.6049 × 10^−4^	2.1537	2.5163 × 10^−7^	4.9942 × 10^−4^
Sports_Hall	LSTM	6.5955 × 10^−4^	1.2397	8.5151 × 10^−7^	9.2237 × 10^−4^
	BiLSTM	6.7640 × 10^−4^	1.2836	8.5660 × 10^−7^	9.2524 × 10^−4^
	BiGRU	4.6952 × 10^−4^	0.9228	4.4041 × 10^−7^	6.6353 × 10^−4^
	ERSO-GRU	3.7218 × 10^−4^	0.6650	2.7051 × 10^−7^	5.1950 × 10^−4^

## Data Availability

The data that support the findings of this study are available from the author upon reasonable request.
